# Fabrication of a highly protective 3D-printed mask and evaluation of its viral filtration efficiency using a human head mannequin

**DOI:** 10.1016/j.ohx.2022.e00314

**Published:** 2022-05-08

**Authors:** Yuki Ohara, Junichi Kanie, Katsutoshi Hori

**Affiliations:** a Friend Microbe Inc., Aichi 464-0858, Japan; b Department of Biomolecular Engineering, Graduate School of Engineering, Nagoya University, Nagoya, Aichi 464-8603, Japan

**Keywords:** Facemask, COVID-19, Aerosol, Droplets, 3D printer

## Abstract

Facemasks are one of the most effective and low-cost prophylactics for COVID-19. In the spring 2020, when a severe shortage of facemasks occurred worldwide, various types of 3D-printed masks were designed and proposed. However, the protective effects conferred by most of these masks were not experimentally evaluated. Here, we provide a new simple design of 3D-printed mask and evaluate its protective effect in a viral filtration test using a human head mannequin. The developed mask can be constructed with a low-cost 3D printer, with an approximate production cost of US $4. This mask has three parts: the main part, wearing parts, and a piece of non-woven fabric filter. The volume of the filter, which needs to be changed daily, was reduced to approximately 1/10 of that of commercially available surgical masks used in this study. The developed mask is fabricated from polylactic acid, a biodegradable plastic, and its surface contour contacting the face may be adjusted after softening the material with hot water at 60–80 °C. The viral filtration efficiency of the developed mask was found to be over 80%. This performance is better than that of commercially available facemasks, such as surgical masks and cloth masks, and equal to those of KN95 and KF94.

## Highlights

• A simple design 3D-printed mask can be constructed at the cost of $4.
• The volume of the filter was reduced to 1/10 of that of surgical masks.
• The protective performance is better than surgical masks and cloth masks.

## Graphical abstract

**Figure:**
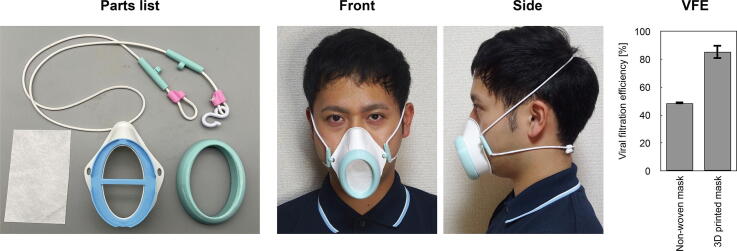

**Specifications table:** 

Hardware name	3D-printed mask
Subject area	General
Hardware type	Personal Protective Equipment
Closest commercial analog	Facemask
Open source license	CC BY
Cost of hardware	US $4
Source file repository	http://dx.doi.org/10.17632/t4rxhrgrt8.1#folder-25fad5ff-a7c5-4704-9999-126b822fdfe1

## Hardware in context

The coronavirus disease 2019 (COVID-19) pandemic caused by SARS-CoV-2 has been forcing us to change our lifestyles to prevent viral infection. Vaccination against SARS-CoV-2 is now underway, and in many countries, the number of infected people is decreasing dramatically [1], [2], [3]. Although vaccination is expected to bring the pandemic to an end, conventional infection prevention measures must be continued until vaccination is widespread. In addition, there are risks of the emergence of mutants that do not respond to available vaccines and that are increasingly virulent. Wearing a facemask is one of the most effective and inexpensive measures to prevent viral infections. When the SARS-CoV-2 pandemic started in March 2020, the effectiveness of facemasks for the prevention of viral infection was unclear, and the guidelines for wearing facemasks differed from country to country [4]. Since then, the effectiveness has been demonstrated both statistically and experimentally [5], [6], [7], [8], [9], [10], [11], [12].

The capacity of facemasks and respirators depends on two factors: the efficiency of the filter material to block (filtrate) particles, and fitting for faces when wearing them. The bacterial filtration efficiency (BFE) and/or particle filtration efficiency (PFE) of the filter material are tested for the former, and leakage is measured using a quantitative fit testing apparatus such as TSI Portacount for the latter in the case of respirators. The facemask is a personal protective equipment used to prevent splashed aerosols and droplets exhaled by wearers [5], [6], [8], [13], [14]. There are many types of facemasks, however, the recommended infection-control masks are surgical masks that meet or exceed the ASTM 2100 standard. The ASTM 2100 standard requires masks to meet criteria such as BFE, PFE, breath resistance, and blood impermeability. Respirators are designed to protect the wearer from aerosols and droplets [13], [14], [15]. The N95 respirator is a standard defined by the National Institute for Occupational Safety & Health (NIOSH) and is required to meet or exceed 95% blocking efficiency against NaCl particles that are 0.3 µm in size. In addition, the respirator needs to fit the wearer’s face to effectively protect the wearer [15]. It is necessary to select an item that fits the wearer individually using a fitting test. Thus, the requirements of surgical masks and respirators differ. The latter are necessary for individuals who come in close proximity to patients with potentially virulent pathogens, but the former are insufficient to protect them against infections [14]. However, since surgical masks are effective in reducing exposure to infectious aerosols, the general public, including asymptomatic individuals, should wear a surgical mask during a pandemic.

On the other hand, during a pandemic, even facemasks are required to have self-protective effects against infection, especially for people who have considerable contact with other people daily. Nevertheless, at the beginning of the COVID-19 pandemic, there was a shortage of both respirators and general surgical masks, as well as the non-woven raw materials themselves [16]. Alternatives such as home-made cloth masks and three-dimensional (3D)-printed masks have been suggested [17], [18], [19], [20], [21]. In addition to the function of general masks (source control), these facemasks were expected to function similarly to respirators (wearer exposure) in some situations [22]. They could not offer the same blocking efficiency as N95 respirators but were expected to decrease the exposure of uninfected wearers to reduce the risk of viral infection [23], [24], [25]. On the other hand, several raw materials were evaluated as alternatives to non-woven fabric filters [24], [26], [27], and cloth masks were revealed to provide only limited protection [28]. However, the performance of 3D-printed masks has never been evaluated in detail [17], [20].

The Japanese Industrial Standard (JIS) T9001 specifies a test method for the viral filtration efficiency (VFE) of the filter material, and the Japanese mask industry association requires that VFE must be tested and indicated as well as BFE and PFE when a facemask claims to be effective in reducing the risk of viral infection. In Japan, numerous commercially available facemasks, including ASTM Level 1–3 surgical masks, advocate that their filters are highly effective at 99% VFE. However, this VFE value is the result of evaluating the filtration capacity of the filter material, typically a non-woven fabric filter, as a flat sheet; that is, leakage is not considered. The actual VFE of common surgical masks is at most 50% when the wearer inhales owing to leakage [8], [15], [23], [29], [30].

Here, we describe the design and assembly of a newly developed 3D facemask fabricated using a 3D printer (Raise3D E2, Raise 3D Technologies, Inc., Irvine, CA, USA). VFE was determined under near-real-use conditions, that is, the 3D mask was worn by a human head mannequin. Having an open-source 3D model of an effective 3D-printable mask could provide a stopgap for future temporary shortages of self-protective masks.

## Hardware description

**Fig. 1:**
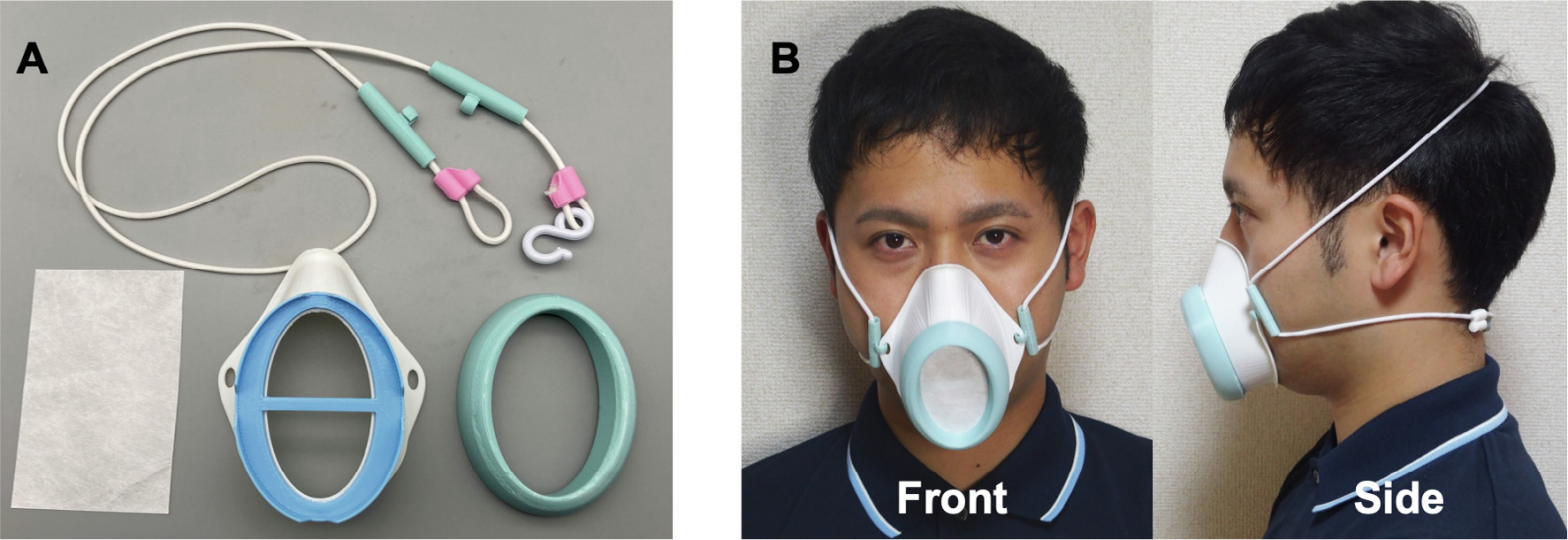
The 3D-printed mask developed in this study (A) and a photo of the worn mask (B).

The new 3D-printed mask consisted of three main parts (base part, filter folder, and cup part), four wearing parts (side part, rubber strap, S-hook, and end part), and a piece of non-woven fabric filter (Fig. 1A). These parts were assembled into a wearable 3D-printed mask, as shown in Fig. 1B. Most of commercially available surgical masks have a three-layer structure consisting of three sheets of non-woven fabric filters and the filter sheets of those examined in this study had a size of 170 mm × 170 mm × 0.21 mm. In contrast, the 3D-printed mask had a monolayer of the filter sheet with a size of 80 mm × 100 mm × 0.21 mm and therefore, it had approximately 1/10 the volume of non-woven fabric used in many commercial surgical masks. The easily adjustable rubber strap attached to the 3D-printed mask can be stretched around the back of the head and neck to secure the mask in place. To fit a large variety of face shapes, the base part is designed to directly touch the contour line connecting the nasal bone, side of the nose, side of the mouth, and bottom of the chin, which is relatively even. The contact surface is rounded to reduce facial irritation. The base part is fabricated from polylactic acid (PLA) resin and is thermally deformable; therefore, the shape can be adjusted by heating with hot water to fit individual faces.

• The new mask consists of three main parts: a main part, wearing parts, and a non-woven fabric filter.
• The volume of the non-woven filter is 1/10 of that of many commercial surgical masks.
• The surface shape contacting the face is adjustable with hot water.
• The new mask realizes over 80% of VFE when worn.

## Design files summary

**Table:** 

**Design file name**	**File type**	**Open source license**	**Location of the file**
Base_part.stl	STL	CC BY	http://dx.doi.org/10.17632/t4rxhrgrt8.1#file-09e60bf1-db7c-4213-ab66-65739cbbbe40
Filter_holder.stl	STL	CC BY	http://dx.doi.org/10.17632/t4rxhrgrt8.1#file-ccc7921c-4528-480f-af5a-3dbcefe3cb5a
Cup_part.stl	STL	CC BY	http://dx.doi.org/10.17632/t4rxhrgrt8.1#file-f07ebd58-71a5-4227-8577-27e05904f977
Side_part.stl	STL	CC BY	http://dx.doi.org/10.17632/t4rxhrgrt8.1#file-9715b212-4417-41e3-9b72-64d812cf2d8c
End_part.stl	STL	CC BY	http://dx.doi.org/10.17632/t4rxhrgrt8.1#file-6c70d770-41b0-49ff-815b-77dbcd137966

## Bill of materials summary

**Table:** 

**Designator**	**Component**	**Number**	Cost per unit-currency*^1^	Total cost -currency*^1^	**Source of materials**	**Material type**
3D printed parts	Base Part	1	US $0.88	US $0.88		Polymer
	Filter Holder	1	US $0.54	US $0.54		Polymer
	Cup part	1	US $0.8	US $0.8		Polymer
	Side part	2	US $0.10	US $0.20		Polymer
	End part	2	US $0.058	US $0.116		Polymer
Purchased parts	Cylindrical magnet	6	US $0.15	US $0.9	https://www.e-sangyo.jp/neo/r/item/neo-d6X1_5.html	Metal
	Round elastic code (φ3mm, 80 cm)	1	US $0.4		https://www.amazon.com/s?k=Round+elastic+code&ref=nb_sb_noss	Polymer
	S-hook	1	US $0.15	US $0.4	https://www.meiwa-sng.com/item/SA-006/	Polymer
	Non-woven fabric filter	1	US $0.2	US $0.15	https://www.amazon.co.jp/dp/B098N42B3Y?ref=myi_title_dp	

*^1^All materials were purchased in Japanese Yen. These costs were converted into US dollars. US $1 = 110 yen.

## Build instructions

**Fig. 2:**
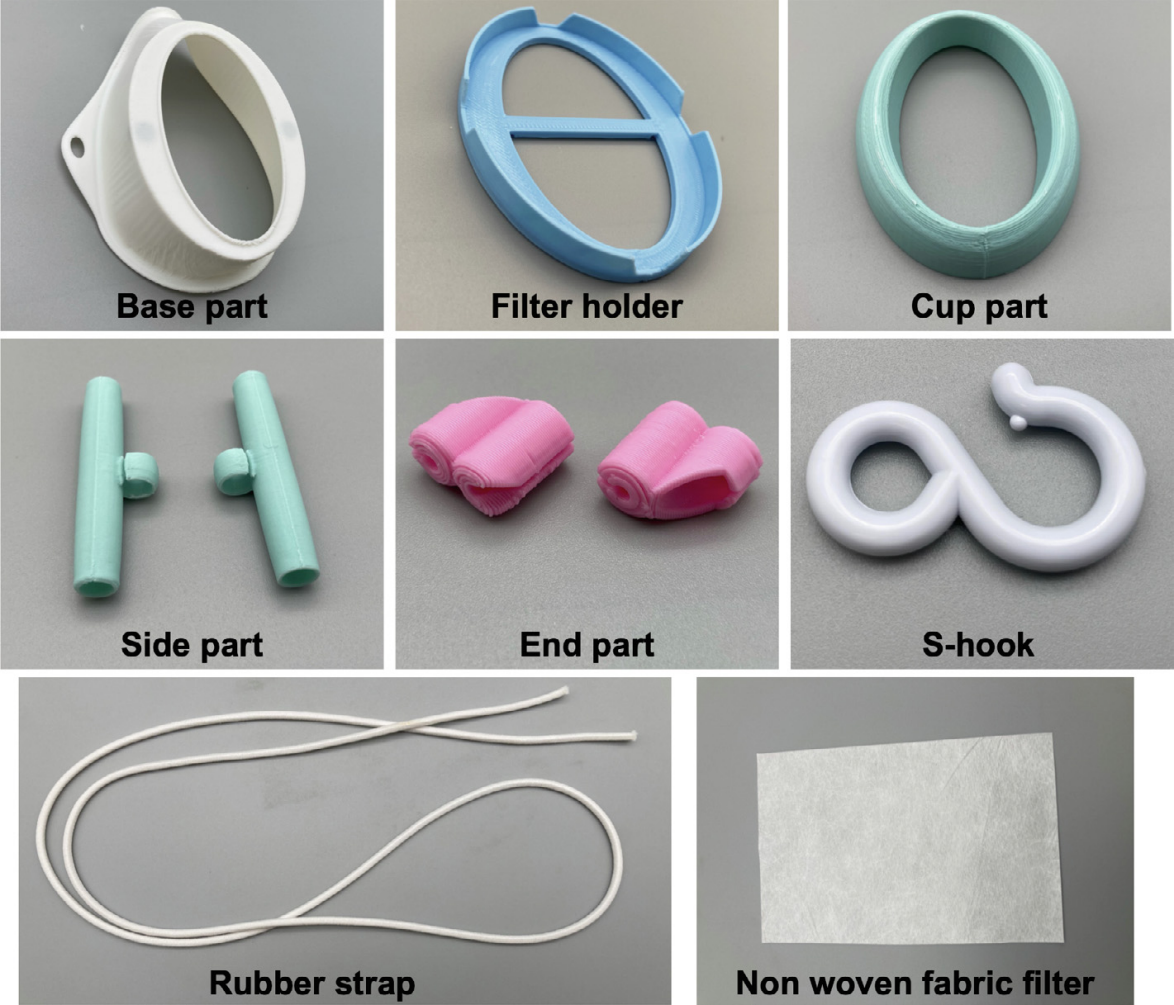
Part list for the construction of the 3D-printed mask.

All parts for assembling the facemasks are shown in Fig. 2, and the construction steps are described below.

### 3-D printing

• The 3D model of the facemask was designed using the open-source 3D creation tool Blender (https://www.blender.org). The 3D model was exported to STL and imported into the software Raise3D ideaMaker (https://raise3d.jp/about).
• PLA with a 1.75 mm diameter was used for printing.
• The speed-E2-PLA preset in ideaMaker was used for slicing. The printing parameters were set as follows: fill density 10%, layer height 0.25 mm, extrusion width 0.40 mm, print speed 60.0 mm/s, extruder temperature 225 °C, and platform temperature 45 °C.
• After printing, the support materials of all parts were removed, and the surfaces were ground using sandpaper (JIS#320, FEPA Grit P280 equivalent).

### Part preparation

**Fig. 3:**
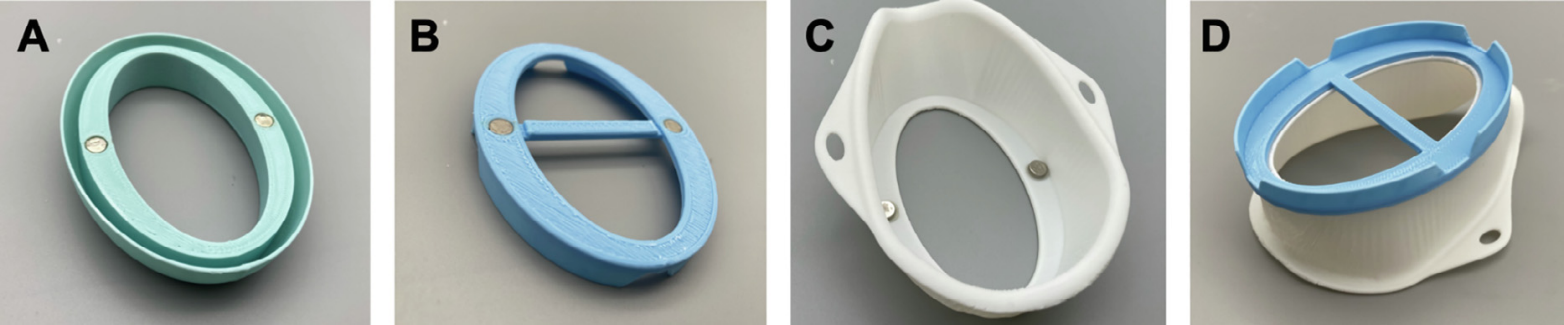
Assembly steps for the base part.
• The filter holder and the base part were glued to each other to avoid generating gaps leading to air leakage (Acrysunday (tetrahydro flan), Acrysunday Co., Ltd., Tokyo, Japan) (Fig. 3D).
• The rubber strap was cut to a length of 80 cm.
• The rubber strap was threaded through the two side parts (Fig. 4A), and a knot was made at each end of the strap (Fig. 4B).

• To enable the assembly of the cup part (Fig. 3A), the filter holder (Fig. 3B), and the base part (Fig. 3C), magnets were fixed onto each pocket using instant glue (CEMEDINE CA-070 3000 Gold Impact Resistant Slim 3 g).

**Fig. 4:**
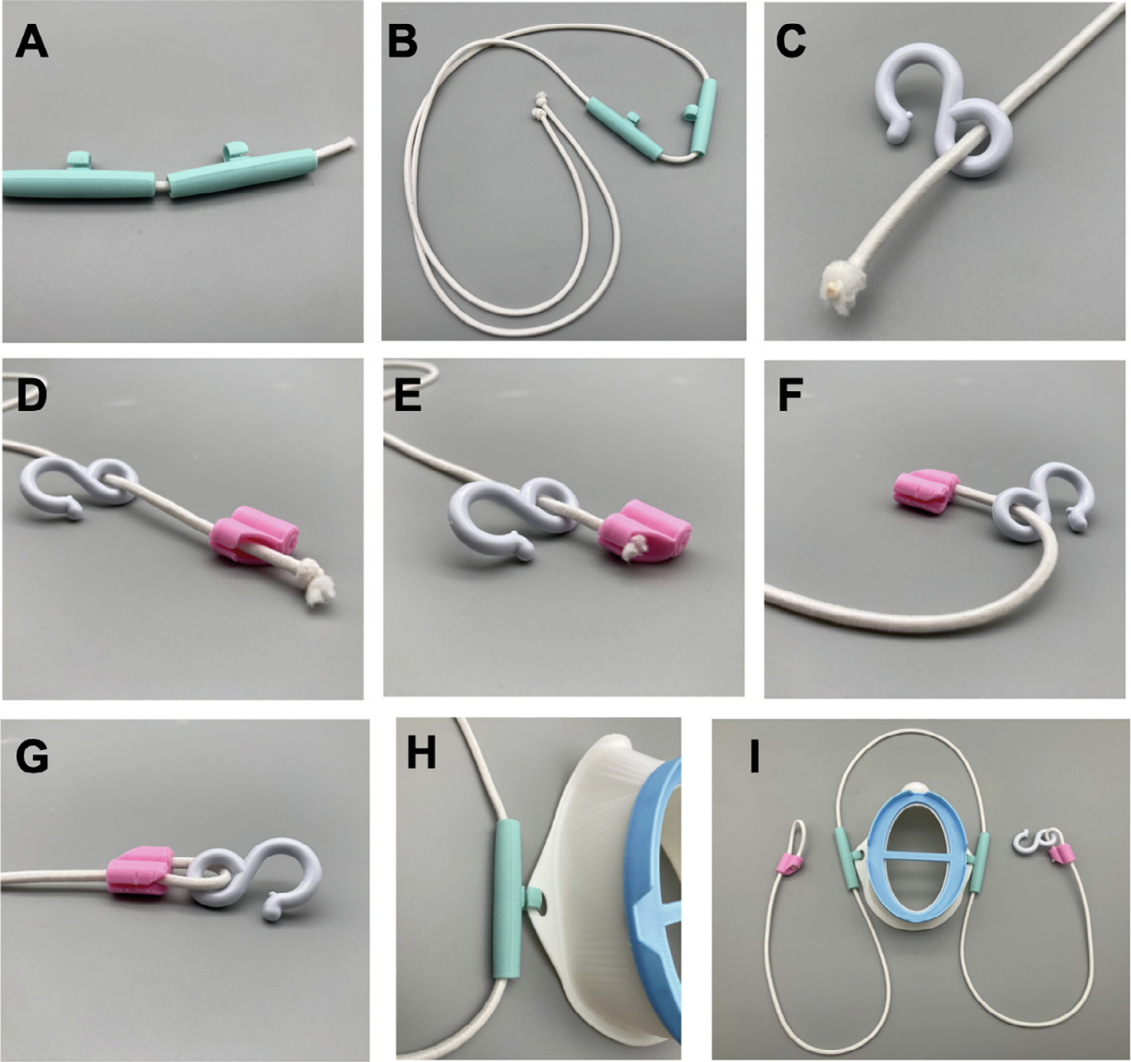
Assembly and attachment of the rubber strap.
• One end of the rubber strap was threaded through the closed hole of the S-hook (Fig. 4C).
• Each end of the rubber strap was passed through the groove of the end part (Fig. 4D). Then, the knot of the string was hidden in the pocket of the end part (Fig. 4E).
• The inner part of the rubber strap was inserted into the groove on the opposite side of the end part, forming a loop that held the S-hook (Fig. 4F, G).
• The opposite end of the rubber strap was assembled in the same manner without the S-hook.
• The hook on each side was inserted into the pinholes at either side of the base part (Fig. 4H), with two ends of the strap dangling downward (Fig. 4I).

### Filter setting

**Fig. 5:**
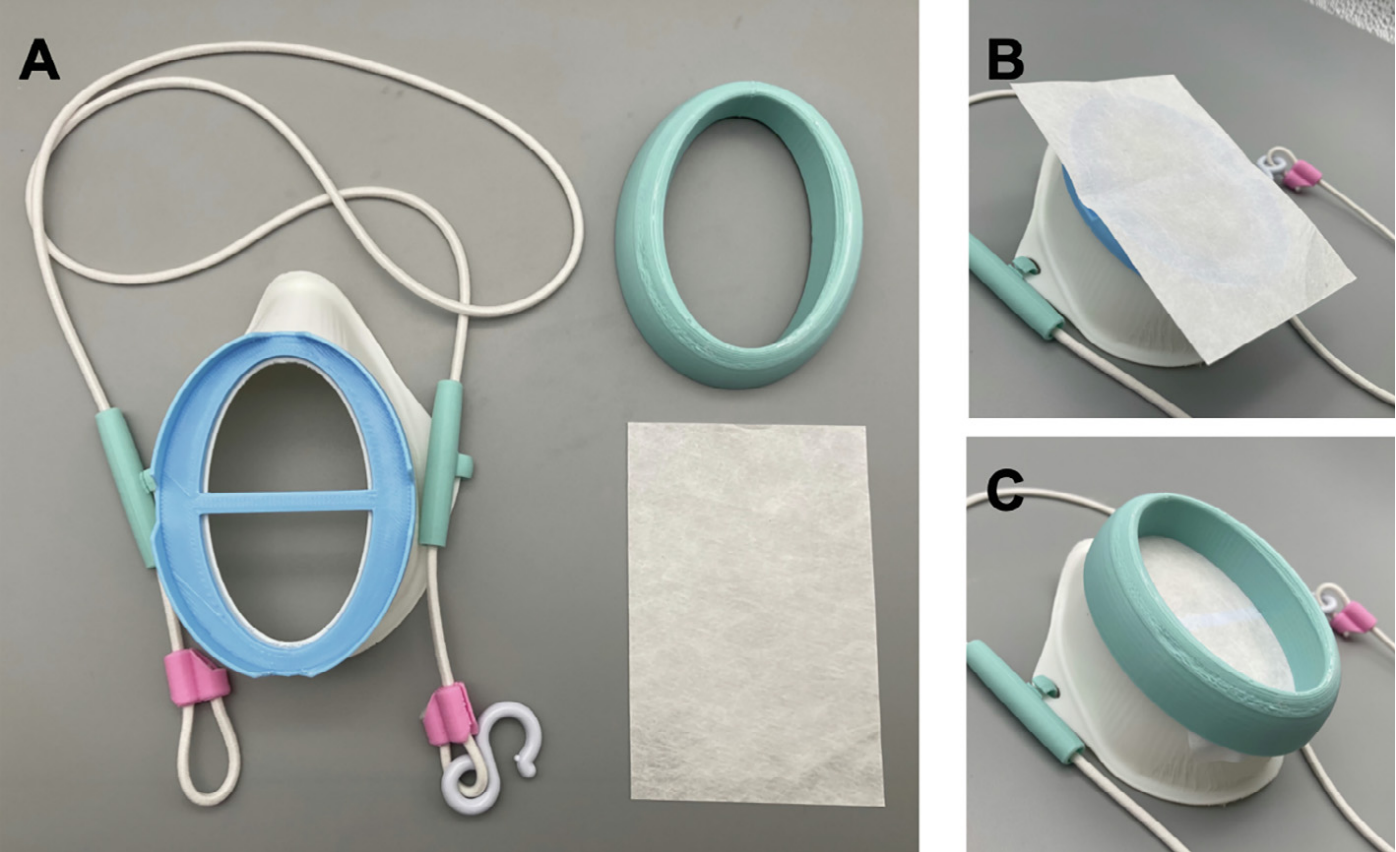
Setting of the non-woven fabric filter onto the 3D-printed masks.
• The non-woven fabric filter was placed on the filter holder assembled as described above (Fig. 5B).
• The cup part was placed onto the filter and pushed so that the convex part around the filter holder was fitted into the groove around the cup part, which was fixed in place by magnets (Fig. 5C).

• A non-woven fabric filter with a VFE of 99% or higher was cut into 80 mm × 100 mm squares (Fig. 5A) *^2^.

*^2^ The non-woven fabric filter used in this study was made by Mitsui Chemicals, Inc. (Tokyo, Japan). The filter was evaluated by Nelson Laboratory (USA) to have a BFE, VFE, and PFE capacity of 99% or higher (Fig. S1). In addition, the VFE was verified to be 99% using the self-made sealed device (Fig. S2), according to JIS T9001 (Table S1). The differential pressure of this non-woven fabric filter was 2.4 mm H_2_O/cm^2^. Other filters with equal capacities can alternatively be used.

## Operation instructions

### Wearing the 3D-printed mask

**Fig. 6:**
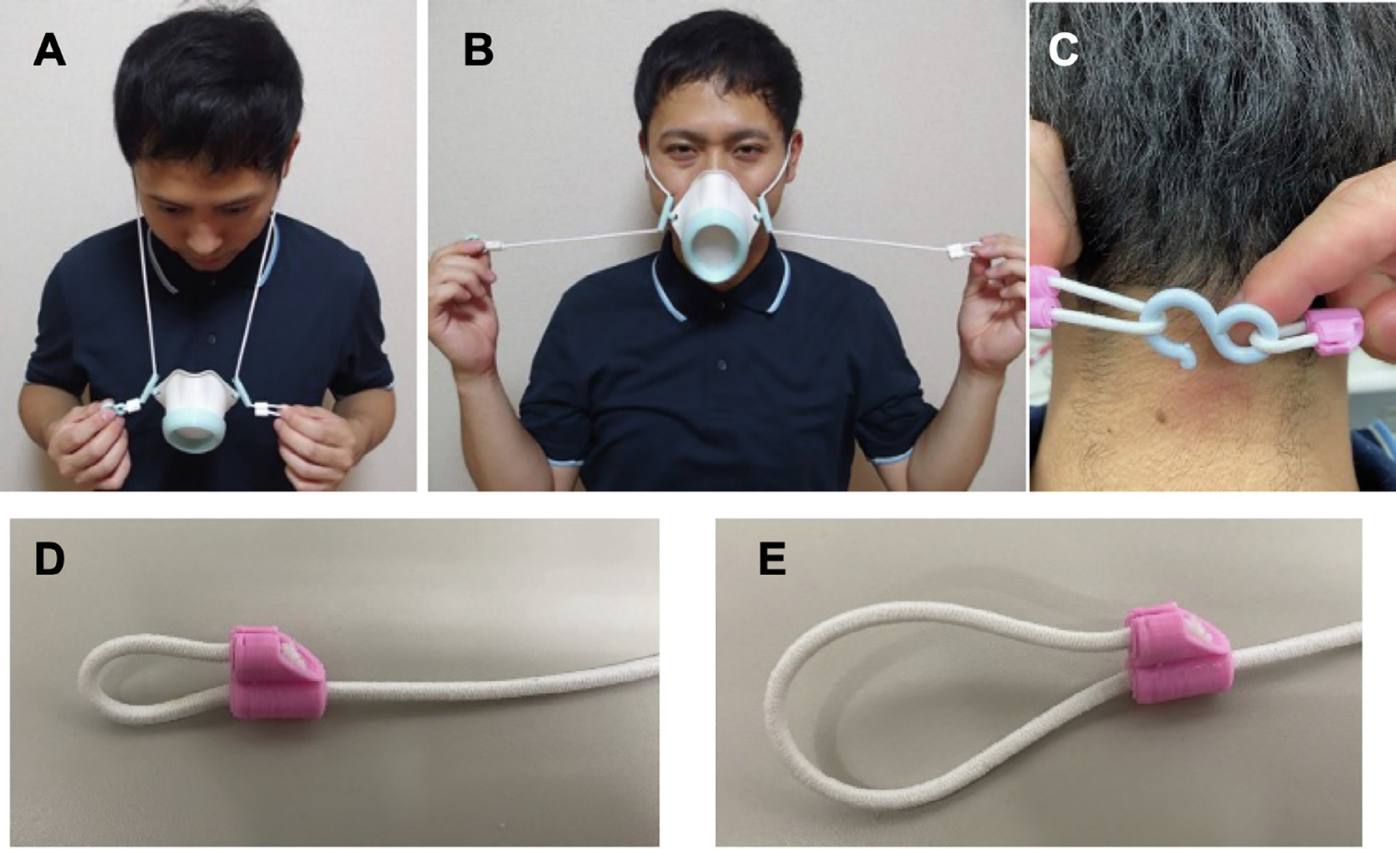
Wearing the 3D-printed mask (A-C) and adjustment of strap length (D, E).
• Both ends of the strap are pulled with both hands (Fig. 6B).
• The loop and hook are held at the back of the neck to hold the mask in position (Fig. 6C).
• The strap length can be adjusted by moving the end-part position (Fig. 6D, E).

• The rubber strap hangs on the back of the head (Fig. 6A).

### Adjusting the contact surface of the 3D-printed mask

**Fig. 7:**
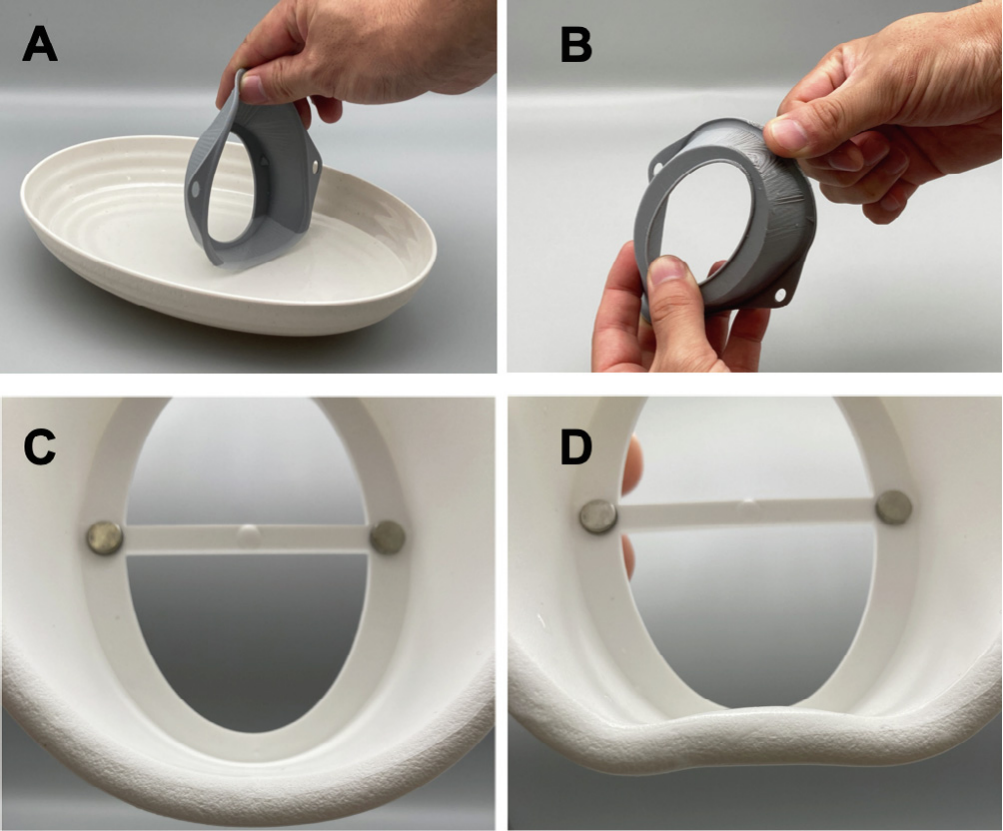
Adjustment of the 3D-printed mask with hot water (A, B). The photos show the 3D-printed masks before adjustment (C) and after adjustment (D).
• After removing it from hot water, the target area is slowly deformed by pressing it with a finger (Fig. 7B).

The gap between the face and contact surface of the 3D-printed mask can be closed using the following steps:

• The base part is soaked in hot water at 60–80 °C for 5–15 s (Fig. 7A).

The 3D-printed mask made from PLA can be deformed, as shown in Fig. 7C and D.

## Validation and characterization

### Evaluating the protective efficiency of the new 3D-printed mask

**Fig. 8:**
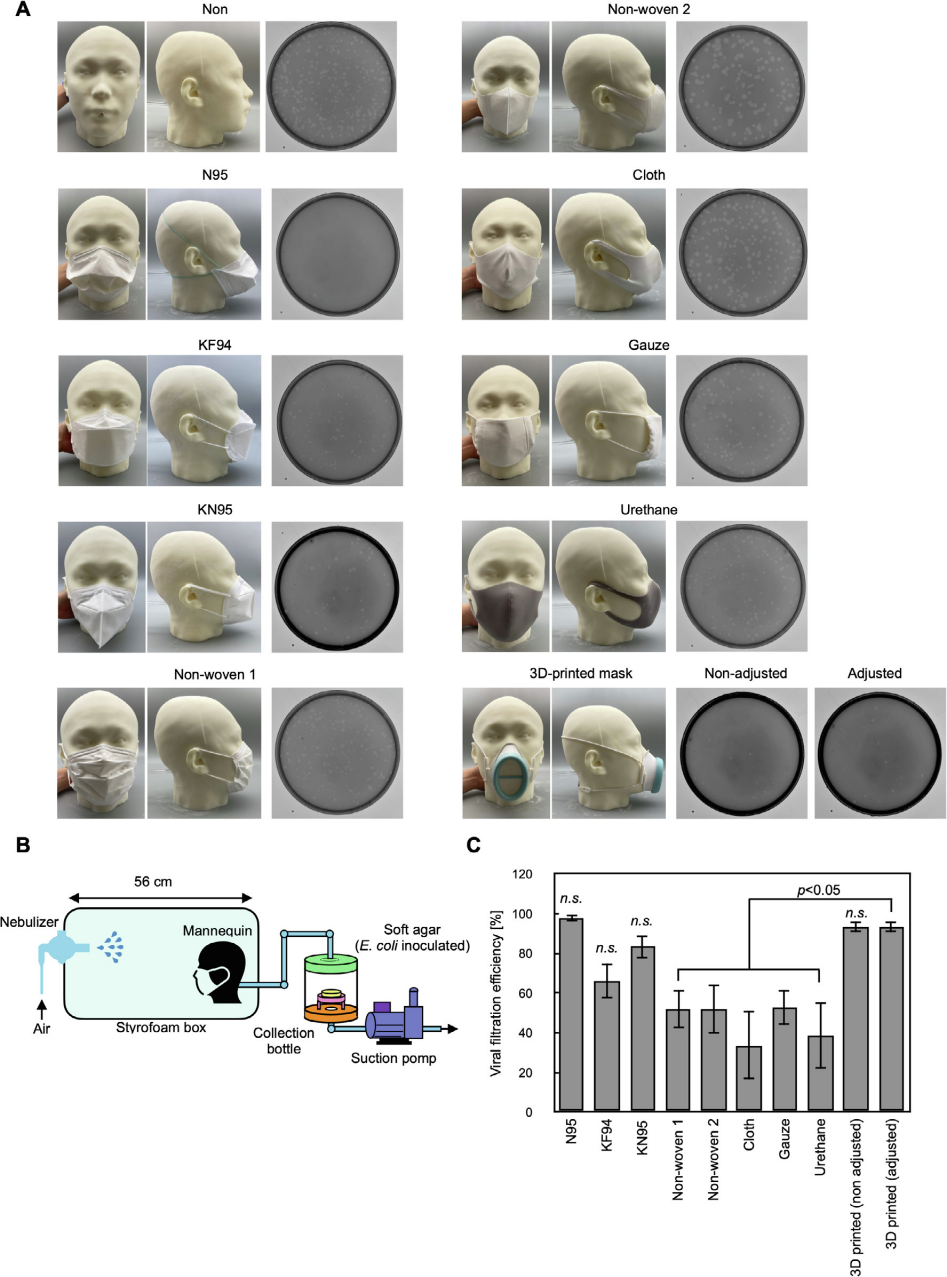
Measurement of the viral filtration efficiency of each facemask and respirator. (A) Photographs of a mannequin wearing each facemask and plaque formed agar plates in the VFE test. (B) The illustration shows the test device for the VFE measurement. (C) Graph of the VFE of each mask. The VFE was calculated from each plaque formation unit. Error bars represent the mean ± SD (N = 6). Statistical significance between 3D printed (adjusted) and other samples was determined by Dannett’s test. n.s., not significant.

The VFE of the 3D-printed mask developed in this study was evaluated using a human head mannequin (Fig. 8A) as previously reported [8]. Other facemasks, non-woven facemask 1 (TOPVALUE Non-woven mask; IEON Co., Ltd., Tokyo, Japan), non-woven facemask 2 (Chourittai standard, Unicharm Corp., Tokyo, Japan), cloth facemask (anti-virus mask, Lion-ya Co., Ltd, Kobe, Japan), gauze facemask (Japanese government provided), and urethane facemask (Best answer 3D urethane mask, Best Answer Co. Ltd., Hyogo, Japan), and respirators, N95 (NafiaS® N-95, NafiaS Inc., Nagano, Japan), KF94 (KLARING, Clean & Science Corp., Seoul, Korea), and KN95 (Suruga Co., Ltd., Shizuoka, Japan), were also evaluated for comparison. The 3D model of the human head mannequin was purchased from DIGITAL HUMAN TECHNOLOGY INC (Japanese average head size model (male version), Kanagawa, Japan), and the test mannequin was constructed using silicone gel (Ascar C0, EXSEAL Co., Ltd. Gifu, Japan), the hardness of which was adjusted to that of the human skin. A plastic pipe (inner diameter = 6 mm) was inserted into the oral cavity of the mannequin head to suck the air. The VFE of the facemasks and respirators were determined using this mannequin head. The bacteriophage φX174 (NBRC103405) and *Escherichia coli* C strain (ATCC13706) were used as model viruses and propagation hosts for the bacteriophage, respectively, according to JIS T9001. To make the bacteriophage form plaques, double-layer soft agar plates were prepared; 20 mL nutrient broth (NB) medium (Bacto™ peptone: 1%, Yeast extract: 0.2%, NaCl: 0.5%, MgSO_4_・7H_2_O: 0.1%) containing 1.5% agar was poured into a petri dish as a lower layer, and as a top agar layer, 3 mL of NB medium containing 0.75% agar and *E. coli* cells at 0.1–0.3 of an optical density at 600 nm was overlayed onto the solidified lower layer.

A VFE test system was constructed using a nebulizer, the mannequin head described above, a suction pump, and a collection bottle, as shown in Fig. 8B. The viral solution was prepared in sodium-magnesium buffer (NaCl; 100 mM, MgSO_4_･7H_2_O; 7 mM, Tris-HCl (pH7.5); 50 mM, gelatin; 0.01%) with a titer of approximately 2.5 × 10^5^ pfu/mL [31], and then transferred to a glass nebulizer (flat type, Ishiyama-Rikagaku Glass Manufacturing Corporation, Tokyo, Japan). Air was injected into the nebulizer at 0.05 MPa for 30 s to generate aerosols and sucked at 25 L/min for 120 s using a suction pump (Suction pump SP40, Tokyo M. I. COMPANY, INC., Tokyo, Japan). The virus particles inhaled from the oral cavity of the head mannequin were captured on the double-layer soft agar plate, placed in the collection bottle, and incubated at 37 °C. The number of phage plaques formed on the plate was counted.

The VFE was calculated from plaque formation unit according to the following formula.

$$VFE\left(\%\right)=100-\frac{\mathrm{Plaque}\;count\;when\;the\;mask\;is\;equipped}{\mathrm{Plaque}\;count\;when\;the\;mask\;is\;not\;equipped}\times 100$$

While the VFEs of conventional facemasks, such as non-woven, cloth, gauze, and urethane facemasks, were only approximately 50%, that of the developed 3D-printed mask was 93 ± 3%, showing a significant difference between the new 3D-printed mask and the conventional facemasks (Fig. 8C). On the other hand, the VFEs of respirators N95, KF94, and KN95 were 98 ± 2%, 67 ± 9%, and 84 ± 6%, respectively, showing no statistically significant difference between these respirators and the new 3D-printed mask in terms of VFE. Adjusting the 3D-printed mask with hot water did not improve the VFE (93 ± 3%). This could be due to the fact that the 3D-printed mask was sufficiently fitted to the mannequin.

### Verification of leakage from worn facemasks

**Fig. 9:**
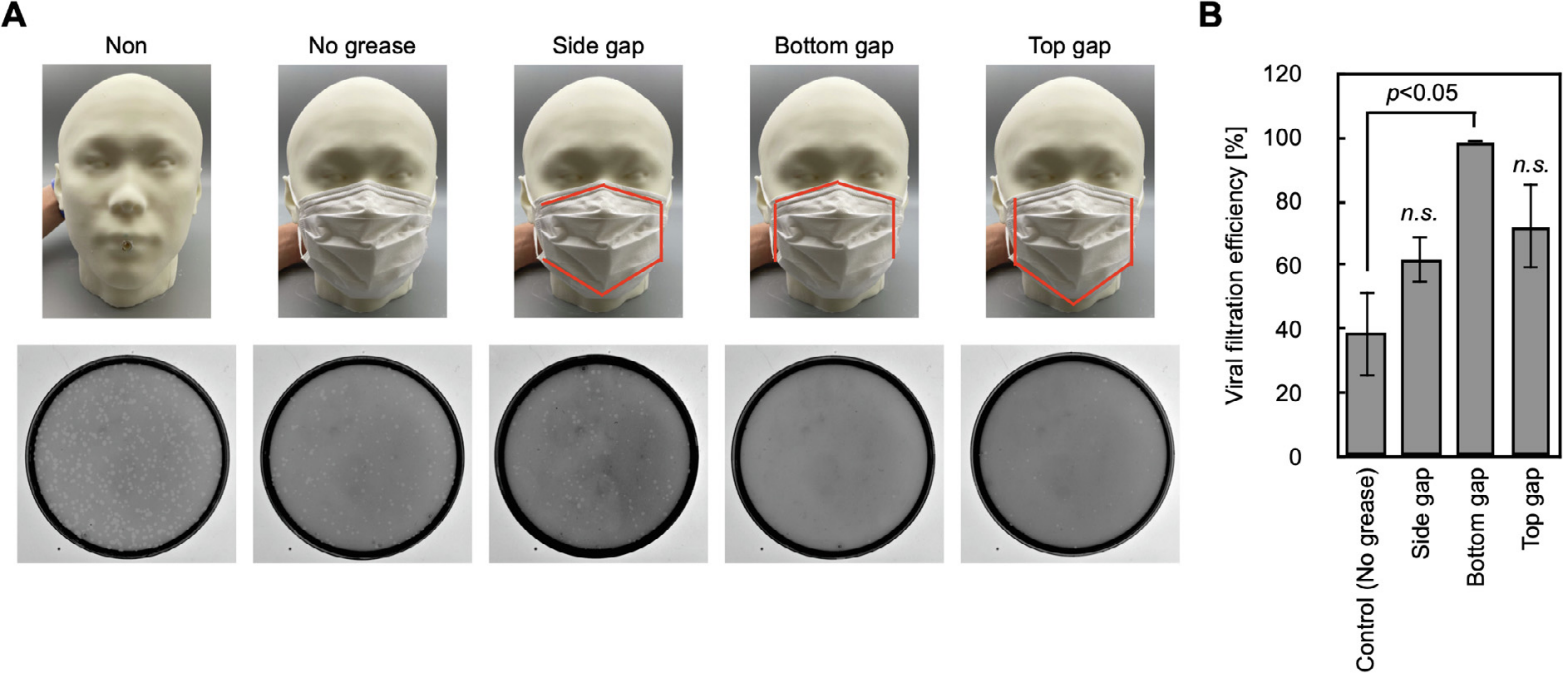
Measurement of leakage caused by the air gap between face and non-woven facemask. The labels “side gap,” “bottom gap,” and “top gap” indicate which gap was left unsealed in each of the VFE tests. (A) Upper photographs are human head mannequins wearing the tested facemask. Red lines indicate the gaps sealed using vacuum grease. Lower photographs are of the plaque-formed agar plates from the VFE test. (B) The graph shows the VFE under each test condition. Error bars represent the mean ± SD (N = 3). Statistical significance between control (No grease) and other samples was determined by Dannett’s test. n.s., not significant.

The two types of non-woven facemasks evaluated in this experiment had VFEs similar to cloth, gauze, and urethane facemasks, in spite of the use of a non-woven fabric filter with a VFE of 99% [26], [27]. These results were thought to be caused by leakage through the gap between the worn facemask and mannequin surface. To confirm this, the gaps along three of the four sides of the non-woven facemask 1 were sealed with vacuum grease (MOLYKOTE® High Vacuum Grease, DuPont Toray Specialty Materials K.K., Tokyo, Japan) (Fig. 9). Thereafter, the VFE test was again performed, as described above. The results showed that leakage from the bottom gap was small, but leakage from the side and top gaps was large (30–40% decrease in VFE).

**Fig. 10:**
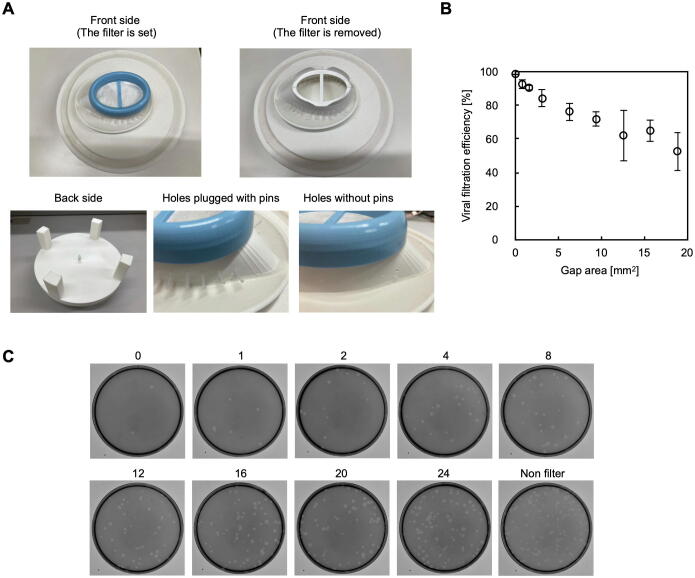
Correlation between the gap and leakage. (A) The photograph shows the test device for adjusting the gap size. The device has 24 holes each with a 1 mm diameter, and the holes are stoppable with pins. (B) The graph shows the VFE used to evaluate the correlation between the gap area and leakage. Error bars represent the mean ± SD (N = 3). (C) The photographs show plaque-formed agar plates. The numbers above each photograph indicate the number of opened holes.

Our 3D-printed mask was used to quantitatively examine the effect of the gap size on the leakage. To simulate the gap, 24 holes with a diameter of 1 mm were drilled into the sides of the mask, and the area of the gap was adjusted by inserting pins and blocking these holes (Fig. 10A). In the VFE test, 0, 1, 2, 4, 8, 12, 16, 20, and 24 holes were opened. This test showed that small gaps of 5 mm^2^ caused a decrease in the VFE of approximately 20% (Fig. 10B, C). The VFE decreased as the gap area increased, reaching a 50% decrease at a gap of 20 mm^2^. Therefore, it is considered that the difference in VFEs between this 3D-printed mask (93%) and its filter material itself (99%) was caused by leakage from the gap between the 3D-mask and the mannequin surface. The leakage of virus particles was much smaller than that of the conventional facemasks examined in this study and was almost at the same level as that of respirators. Hence, our 3D-printed mask is expected to reduce the risk of virus infection for the wearer more than general surgical masks.

### Quantification of the residual virus on the filter and the base part of the 3D-printed mask

**Fig. 11:**
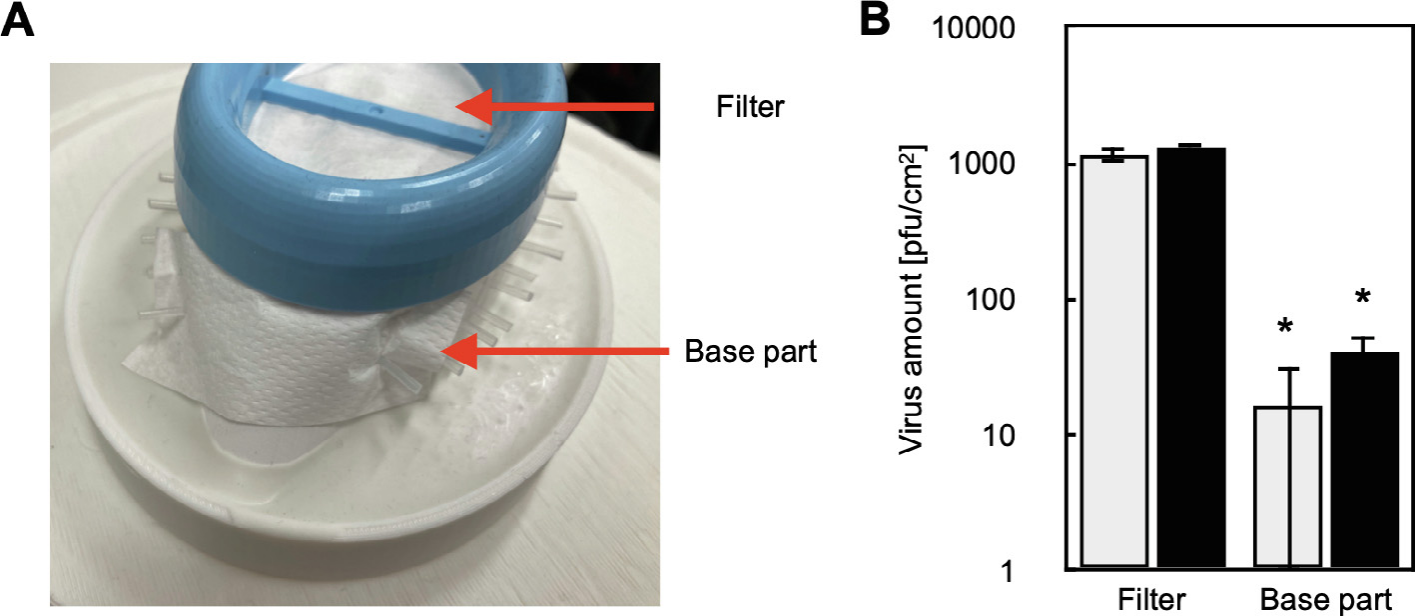
Quantification of the amount of virus attached to the filter and base part during the VFE test. (A) Quantification targets. The mask filter and base part. A piece of filter was attached to the side of the base part to collect residual virus. (B) Quantification of the residual virus. The black and white bars show the titer per area calculated from qPCR and plaque formation units, respectively. Error bars represent mean ± SD (N = 3). * indicates significant differences determined by *t*-test.

Virus particles can adhere to the surface of the facemask during use. Therefore, after each VFE test was performed, the residual virus on the non-woven fabric filter and the base part was quantified by qPCR and plaque counting (Fig. 11). To do this, an additional non-woven fabric filter sheet was lain over the surface of the base part (Fig. 11A) before the VFE test. After the VFE test, each non-woven fabric was transferred into a 50 mL polypropylene centrifuge tube. Twenty milliliters of deionized water was added to the tube and mixed vigorously to dislodge the virus particles from the fabric. The solution was mixed with NB soft agar containing *E. coli* for plaque counting, as described above (7.1). The detached viral solution was mixed with qPCR Master Mix (Luna Universal qPCR Master Mix, New England Bio Labs, Inc., Ipswich, MA) containing the primer set targeting the φX174 genome (forward primer: GCAGGAATTACTACTGCTTGTTTACG, reverse primer; GTGCCAAGCATATTAAGCCAC). qPCR was performed using Step One Plus™️ (Applied Biosystems, Waltham, MA, USA), and the Ct value was determined. Viral titers per area were calculated from Ct values using a calibration curve. Both qPCR and plaque counting revealed that the number of residual viruses was 10–100 pfu/cm^2^ on the surface of the base part and over 10^3^ pfu/cm^2^ on the mask filter (Fig. 11B). Because the quantity of residual virus particles on the mask filter was almost 100 times that on the base part, care must be taken to avoid touching the mask filter directly, and/or without a cleaning agent, when replacing it.

## Discussion

In this study, we demonstrated an easy and simple method for assembling a new 3D-printed mask and evaluated its protective effect against viruses through the VFE test. The developed mask had a better protective effect than commercially available facemasks, such as non-woven, cloth, and polyurethane facemasks. The release of open-source data on how to construct a performance-assured facemask would be helpful for preventing another shortage of effective facemasks, as during the beginning of the COVID-19 pandemic. Currently, the global supply of facemasks is stable; the shortage of facemasks around the spring of 2020 has been completely resolved. Moreover, as vaccination progresses, it is expected that wearing facemasks will become unnecessary in the near future. However, later in the future, when aerosol-mediated infectious diseases become prevalent again, our experience with COVID-19 control will undoubtedly lead to the use of facemasks for initial prevention. Storing open-source models of 3D-printed masks with guaranteed performance will be an effective way to prepare for unknown infectious diseases in the future.

The total cost of the materials used to produce a 3D-printed mask is US $4. As shown in Fig. 11, since the viruses remained in the PLA part, it is necessary to clean this part. The lifetime of plastics is expected to be several years [32]. The cost of our 3D-printed mask is $0.21 per day, assuming that the lifetime of the 3D-printed parts is one year and that the filter is replaced with a new one every day. This cost is much lower than that of respirators (N95, KF94, and KN95) and general surgical masks (non-woven masks) (Table 1).

**Table 1:** Daily cost of each type of mask.

Mask type	Daily cost ($/day)
3D-printed mask*^1^	$0.21
N95*^1^	$1.51
KF94*^1^	$0.64
KN95*^1^	$0.98
Non-woven facemask*^2^	$0.45

Each cost was calculated assuming that one mask was consumed per day. These costs were converted to US dollars. US $1 = 110 yen.

*^1^ Unit price average was calculated by examining the sales prices of more than nine brands as of February.

*^2^ Reference to data published by the Ministry of Internal Affairs and Communications, Japan [36].

Since the COVID-19 pandemic, the amount of waste from facemasks has increased. It is estimated that 129 billion facemasks are discarded each month [33], and waste from facemasks is currently found in the environment, including the oceans [11], [34]. Considering that non-woven masks are made from plastics such as polypropylene and polystyrene [35], there is a concern that the use of facemasks will cause new environmental problems, such as ocean pollution by plastics. The 3D-printed mask developed in this study uses 1/10 the volume of non-woven fabric found in commonly available surgical masks and employs PLA, biodegradable plastic, for the main reusable part. This new 3D-printed mask is expected to effectively prevent infectious diseases and protect the environment at the same time.

## Limitations

The newly developed 3D-printed mask exhibited high performance in the VFE test using a mannequin. However, this mannequin was created based on the shape of the face of a standard Japanese male. Thus one cannot assume that it would fit every face shape. In this 3D-printed mask, leakage caused by facial movements, such as sneezing and talking, was not considered. Although it is believed that some degree of adjustment to individual faces is possible through deformation of the mask by hot water, there may be limitations. The 3D-printed mask was developed as a new facemask. However, the VFE of this mask demonstrated as high as those of the KN95 and KF94 respirators. It is expected to reduce the risk of viral infection by blocking aerosols and droplets inhaled by the wearers. Respirators, on the other hand, must completely fit the face, which is essential to protect the wearer [8], [15]. When using our 3D-printed mask as a substitute for respirators in situations where the risk of infection is high, fit testing with specialized equipment such as the TSI Portacount is essential.

## Additional data

A difference in the size of the base part (large size) was also uploaded: http://dx.doi.org/10.17632/t4rxhrgrt8.1#file-1c19e80c-69a1-42fa-b47f-296330b8e900

## CRediT authorship contribution statement

**Yuki Ohara:** Conceptualization, Validation, Investigation, Writing – original draft, Visualization. **Junichi Kanie:** Methodology, Validation, Visualization. **Katsutoshi Hori:** Conceptualization, Writing – review & editing, Visualization, Supervision, Project administration.

## Declaration of Competing Interest

The authors declare that they have no known competing financial interests or personal relationships that could have appeared to influence the work reported in this paper.
