# Supplementary material for: Fabrication of a highly protective 3D-printed mask and evaluation of its viral filtration efficiency using a human head mannequin
Source: HardwareX. 2022 May 8;11:e00314. doi: 10.1016/j.ohx.2022.e00314 (PMC9078936; doi:10.1016/j.ohx.2022.e00314)
Supplement: Supplementary data 1 [file mmc1.docx]

**Supplementary Figures for**

Fabrication of a highly protective 3D-printed mask and evaluation of its viral filtration efficiency using a human head mannequin

Yuki Ohara, Junichi Kanie, Katsutoshi Hori


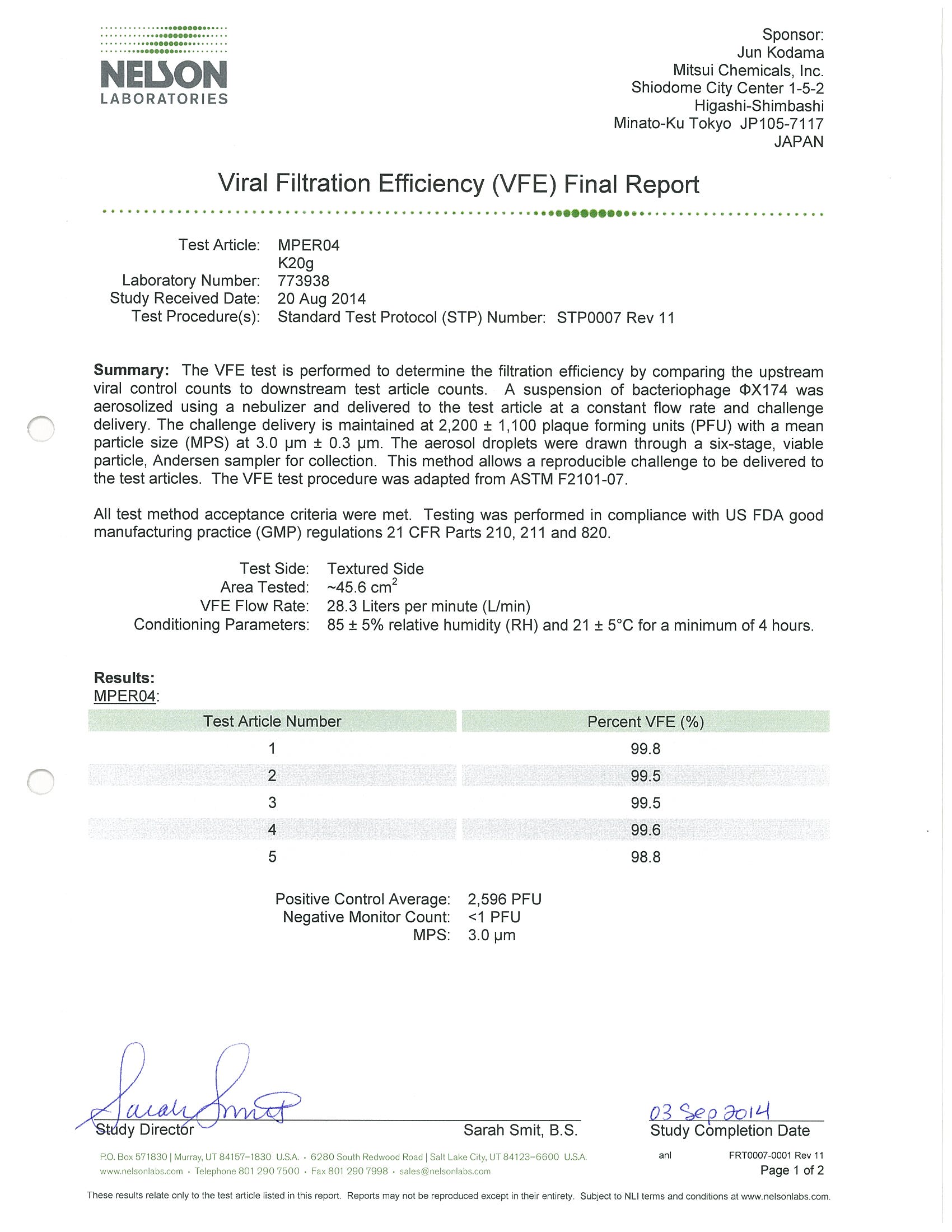


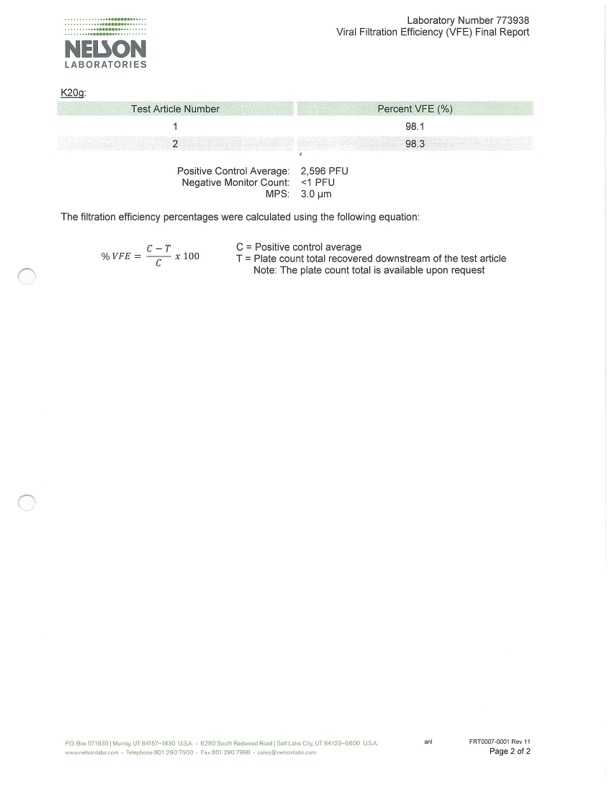


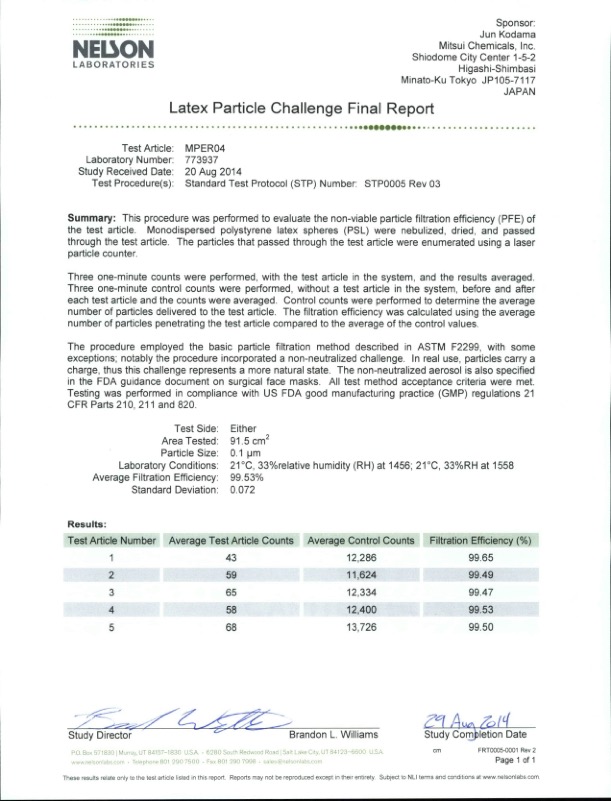


**
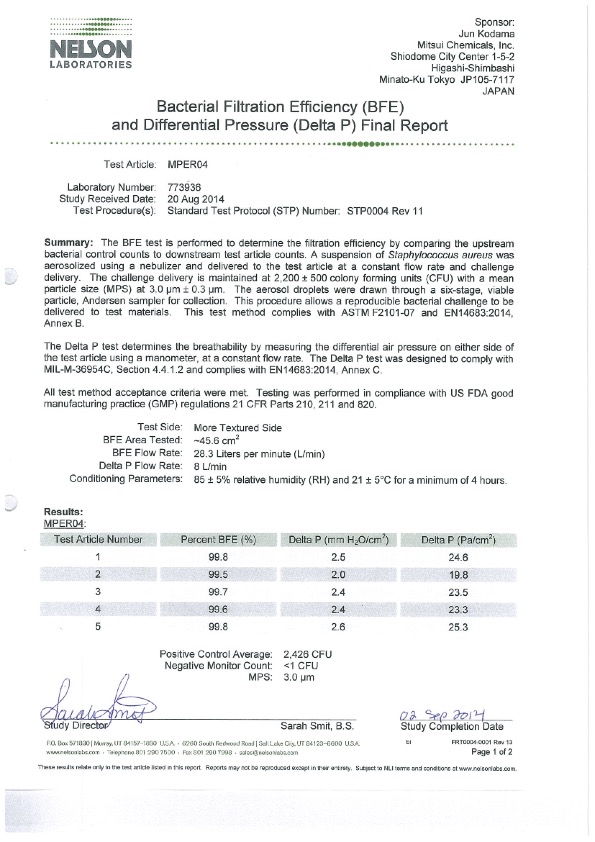
**

Figure S1 The filtration efficiency and differential pressure reports against non-woven fabric filter from nelson laboratories.

Figure S2 Viral filtration efficiency test against non-woven fabric filter. (A) The illustration shows the test device for the VFE measurement against filter. (B) The photograph shows the filter setting.
