# Supplementary material for: Fabrication of a highly protective 3D-printed mask and evaluation of its viral filtration efficiency using a human head mannequin
Source: HardwareX. 2022 May 8;11:e00314. doi: 10.1016/j.ohx.2022.e00314 (PMC9078936; doi:10.1016/j.ohx.2022.e00314)
Supplement: Supplementary data 2 [file mmc2.docx]

**Supplementary Tables for**

Fabrication of a highly protective 3D-printed mask and evaluation of its viral filtration efficiency using a human head mannequin

Yuki Ohara, Junichi Kanie, Katsutoshi Hori

**Table S1 Figure S2 Viral filtration efficiency test against non-woven fabric filter**

| Control | Filter |
| --- | --- |
| 271 | 1 |
| 290 | 1 |
| 336 | Undetected |
|  | Undetected |
|  | Undetected |

Each number shows the plaque counts. Control means non filter. Control sample conducted three times. Filter sample test was conducted five times. Undetected indicates that no plaque was formed.

**Table S2 Plaque counts of the viral filtration efficiency of each mask and respirators**

| Non | N95 | KF94 | KN95 | Non-woven 1 | Non-woven 2 | Cloth | Gauze | Urethane | 3D-printed (non adjusted) | 3D-printed (adjusted) |
| --- | --- | --- | --- | --- | --- | --- | --- | --- | --- | --- |
| 349 | 1 | 149 | 93 | 175 | 177 | 257 | 176 | 280 | 32 | 27 |
| 284 | 1 | 93 | 56 | 169 | 151 | 227 | 180 | 291 | 17 | 26 |
| 393 | 1 | 160 | 70 | 171 | 138 | 206 | 206 | 191 | 18 | 13 |
| 462 | 12 | 133 | 60 | 221 | 189 | 274 | 159 | 160 | 38 | 23 |
| 404 | 14 | 109 | 36 | 163 | 183 | 195 | 175 | 246 | 21 | 22 |
| 351 | 11 | 112 | 51 | 175 | 243 | 328 | 167 | 209 | 23 | 41 |

Each number shows the plaque counts corresponding to Figure8 C. Non means

**Table S3 Measurement of leakage caused by the air gap between face and non-woven facemask**.

| Non | Control no grease | Side gap | Bottom gap | Top gap |
| --- | --- | --- | --- | --- |
| 518 | 406 | 227 | 6 | 109 |
| 737 | 429 | 261 | 12 | 158 |
| 648 | 333 | 237 | 6 | 263 |

Each number shows the plaque counts corresponding to Figure9 B

**Table S4 Correlation between the gap and leakage.**

|  | Non | 0 hole | 1 hole | 2 holes | 4 holes | 8 holes | 12 holes | 16 holes | 20 holes | 24 holes |
| --- | --- | --- | --- | --- | --- | --- | --- | --- | --- | --- |
| 1st | 505 | 8 | 40 | 46 | 60 | 105 | 116 | 104 | 143 | 171 |
| 2nd | 616 | 5 | 61 | 68 | 135 | 183 | 192 | 303 | 221 | 325 |
| 3rd | 174 | 2 | 7 | 14 | 24 | 35 | 53 | 76 | 71 | 96 |

Each number shows the plaque counts corresponding to Figure10 B. Three independent experiments were conducted for this test. The number of plaques in each was converted to VFE for each experiment before the average was calculated.

**Table S5 Quantification of the amount of virus attached to the filter and base part during the VFE.**

| **Filter** | **Base part** |
| --- | --- |
| 123 | 4 |
| 138 | 0 |
| 161 | 2 |

Each number shows the plaque counts corresponding to Figure11 B. Each count was normalized by area for calculation of pfu/cm^2^ in figure 11. The filter area is 24 cm^2^. Base part filter area is 18 cm^2^.
